# Prolonged carriage of ESBL-producing enterobacterales and potential cross-transmission among residents in geriatric long-term care facilities

**DOI:** 10.1038/s41598-021-01190-w

**Published:** 2021-11-03

**Authors:** Ryusuke Ae, Teppei Sasahara, Akio Yoshimura, Koki Kosami, Shuji Hatakeyama, Kazumasa Sasaki, Yumiko Kimura, Dai Akine, Masanori Ogawa, Kenji Hamabata, Longzhu Cui

**Affiliations:** 1 Division of Public Health, Center for Community Medicine, Jichi Medical University, Yakushiji 3311-1, Shimotsuke, Tochigi 329-0498 Japan; 2 Division of Clinical Infectious Diseases, School of Medicine, Jichi Medical University, Yakushiji 3311-1, Shimotsuke, Tochigi 329-0498 Japan; 3 Division of Bacteriology, School of Medicine, Jichi Medical University, Yakushiji 3311-1, Shimotsuke, Tochigi 329-0498 Japan; 4 Medical Corporation Sanikukai Nissin Hospital, Kiryu, Gunma 376-0001 Japan; 5 Division of Infectious Diseases, Jichi Medical University Hospital, Yakushiji 3311-1, Shimotsuke, Tochigi 329-0498 Japan; 6 Clinical Microbiology Laboratory, Jichi Medical University Hospital, Yakushiji 3311-1, Shimotsuke, Tochigi 329-0498 Japan; 7 Health Service Center, Jichi Medical University, Yakushiji 3311-1, Shimotsuke, Tochigi 329-0498 Japan; 8 Gerontological Nursing, School of Nursing, Jichi Medical University, Yakushiji 3311-1, Shimotsuke, Tochigi 329-0498 Japan

**Keywords:** Health care, Medical research, Antimicrobials, Bacteria

## Abstract

Previous studies indicated residents in geriatric long-term care facilities (LTCFs) had much higher prevalence of extended-spectrum β-lactamase-producing Enterobacteriaceae (ESBL-E) carriage than the general population. Most ESBL-E carriers are asymptomatic. The study tested the hypothesis that residents with ESBL-E carriage may accumulate inside geriatric LTCFs through potential cross-transmission after exposure to residents with prolonged ESBL-E carriage. 260 residents from four Japanese LTCFs underwent ESBL-E testing of fecal specimens and were divided into two cohorts: Cohort 1,75 patients with ≥ 2 months residence at study onset; Cohort 2, 185 patients with < 2 months residence at study onset or new admission during the study period. Three analyses were performed: (1) ESBL-E carriage statuses in Cohort 1 and Cohort 2; (2) changes in ESBL-E carriage statuses 3–12 months after the first testing and ≥ 12 months after the second testing; and (3) lengths of positive ESBL-E carriage statuses. Compared with the residents in Cohort 1, a significantly larger proportion of residents in Cohort 2 were positive for ESBL-E carriage (28.0% in Cohort 1 vs 40.0% in Cohort 2). In the subsequent testing results, 18.3% of residents who were negative in the first testing showed positive conversion to ESBL-E carriage in the second testing, while no patients who were negative in the second testing showed positive conversion in the third testing. The maximum length of ESBL-E carriage was 17 months. The findings indicated that some residents acquired ESBL-E through potential cross-transmission inside the LTCFs after short-term residence. However, no residents showed positive conversion after long-term residence, which indicates that residents with ESBL-E carriage may not accumulate inside LTCFs. Practical infection control and prevention measures could improve the ESBL-E prevalence in geriatric LTCFs.

## Introduction

With the rapid aging of populations worldwide, the number of older adults requiring residence in geriatric long-term care facilities (LTCFs) is increasing. For older residents in geriatric LTCFs, outbreaks of specific infectious diseases inside their facilities can greatly affect their mortality and morbidity^1–6^. Therefore, appropriate infection prevention and control is a critical challenge for care providers in geriatric LTCFs^1–6^.

Multidrug-resistant Gram-negative organisms represent an ongoing threat to global public health and necessitate the implementation of practical infection prevention and control guidelines in daily practice and caregiving^7–10^. Among these organisms, extended-spectrum β-lactamase-producing Enterobacterales (ESBL-E) has become widespread in hospital settings as well as geriatric LTCFs worldwide^10–17^. The intestinal tract provides an ideal reservoir for ESBL-E, and carriers of ESBL-E are typically asymptomatic^18–22^. Several studies have indicated that asymptomatic carriers of ESBL-E may require effective surveillance and specific control programs to prevent the infection becoming widespread^21,22^.

The present study focused on asymptomatic LTCF residents with carriage of ESBL-E identified in their feces. In previous studies, LTCF residents had much higher prevalence of ESBL-E carriage than the general population^12,13^. We hypothesized that in geriatric LTCFs, asymptomatic residents with ESBL-E carriage may accumulate through potential cross-transmission after exposure to residents with prolonged ESBL-E carriage. To test this hypothesis, we performed multiple ESBL-E testing with long-term follow-up for continuous residents in LTCFs to assess (1) the proportion of residents who acquired ESBL-E through potential cross-transmission inside the facilities and (2) the length of ESBL-E carriage among ESBL-E-positive residents.

## Methods

### Study settings and participants

We conducted a cohort study among residents receiving long-term care in 4 geriatric LTCFs in Japan. The facilities were selected because they had secure linkage with their own specific back-up hospitals where residents were typically transferred for any medical needs. All 4 LTCFs were anonymized in accordance with the ethics protocol of the study. Brief profiles of the facilities are shown in Table 1.

**Table 1:** Background characteristics of the study settings.

Characteristics	Geriatric long-term care facilities (anonymized)			
	A	B	C	D
Facility type	HSF	HSF	SNH	HSF
Resident capacity	100	50	60	150
Male : Female residents	47 : 53	11 : 32	19 : 50	33 : 67
Age of residents, median (range), year	84 (59–106)	91 (77–105)	87 (70–106)	85 (53–105)
Proportions of residents requiring diapers for excretion	52%	84%	46%	96%
Population density of municipality where facility is located (persons/km^2^)^†^	389	54	389	13,370
Region of Japan	Eastern	Eastern	Eastern	Western
Number of beds in the back-up hospital	90	100	90	327

HSF, health services facility; SNH, special nursing home.

^†^Calculated using the populations of the municipalities in 2019 or 2020.

The study participants were 260 older adults residing in the 4 LTCFs who underwent ESBL-E carriage testing during the study period (August 2018 through March 2020). Residents who did not undergo ESBL-E testing were excluded from the study. Background data for the 260 residents, such as age, sex, and general condition, were not obtained because of the ethics protocol employed in the study.

In Japan, geriatric LTCFs are classified into two main types: (1) geriatric health services facilities and (2) geriatric special nursing homes. The former are intermediate facilities between hospitals and nursing homes, with a primary focus on rehabilitation. These facilities typically have a goal of returning patients to home-based care, although some residents may require long-term care for years. The latter provide daily life support, including end-of-life care^23–25^. The study included three geriatric health services facilities and one geriatric special nursing home.

### Study design

**Figure 1:**
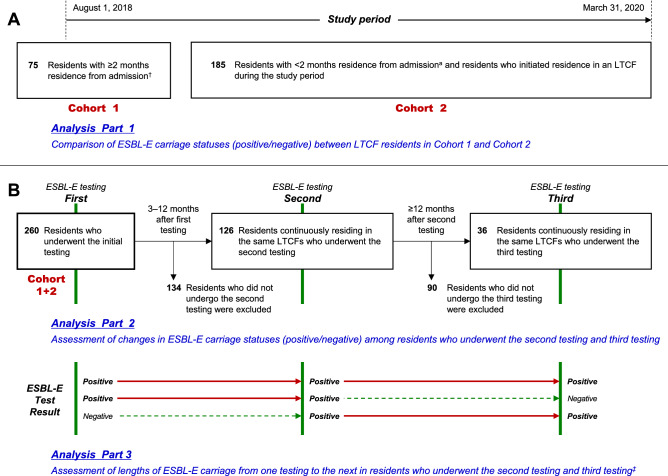
Study design and participants (N = 260). ^†^At the start of the study. ^‡^If all three tests revealed positive results, the lengths of ESBL-E carriage were measured from the first testing through third testing. LTCF, long-term care facility; ESBL-E, extended-spectrum β-lactamase-producing Enterobacterales.

The first ESBL-E testing was performed on all 260 residents < 1 month after study onset or initiation of residence. The 260 residents who underwent the first ESBL-E testing were classified into two cohorts (Fig. 1A): Cohort 1 contained residents who had been residing in an LTCF for ≥ 2 months at study onset, and Cohort 2 contained residents who had been residing in an LTCF for < 2 months at study onset or initiated residence in an LTCF during the study period. First, we compared the ESBL-E carriage statuses (positive/negative) between residents in Cohort 1 and Cohort 2 (Fig. 1A, Analysis Part 1).

Second, we assessed the changes in ESBL-E carriage statuses in residents from the first testing who subsequently underwent a second testing and/or a third testing (Fig. 1B, Analysis Part 2). The second testing was performed 3–12 months after the first testing and the third testing was conducted ≥ 12 months after the second testing for residents who had been continuously residing in the LTCFs. The second testing and third testing were not performed in residents who had been discharged from the LTCFs at the time of each testing. In this analysis, we identified the proportions of residents who had changed from negative to positive for ESBL-E carriage status in the results for the second testing and third testing.

Third, we assessed the lengths of ESBL-E carriage from one testing to the next testing among residents who underwent the second testing and third testing (Fig. 1B, Analysis Part 3). In this analysis, we measured the durations of positive test results in the same residents. If all three tests revealed positive results, the lengths of ESBL-E carriage were measured from the first testing through third testing. The range (minimum to maximum) for the lengths of ESBL-E carriage were determined.

### Microbiology and ESBL-E isolates

Small amounts (0.05–0.1 g) of freshly voided feces from the residents were obtained from paper diapers or papers for self-collection of stool samples (AS ONE Corporation, Osaka, Japan) using stool collection tubes with agar medium (FECES COLLECTING TUBE™; Eiken Chemical Co. Ltd., Tokyo, Japan). To avoid contamination, facility staff performed standard preventive measures during sample collection. Each fecal sample was directly harvested on selective screening agar plates for ESBL-E (CHROMagar™ ESBL/CHROMagar™ mSuperCARBA bi-plate medium [Kanto Chemical Co. Inc., Tokyo, Japan]) using sterile cotton swabs^26^. This medium was able to grow ESBL-E simultaneously with carbapenem-resistant Enterobacteriaceae (CRE) and/or AmpC β-lactamase-producing bacteria. Following aerobic incubation at 37 °C for 18–24 h, colonies growing on either side of the adapted screening agar were regarded as suspect for any of the above bacteria, and underwent further analysis. ESBL-E and AmpC β-lactamase-producing bacteria were identified using an ESBL + AmpC Detection Set™ (MAST Group, Bootle, Merseyside, UK). CRE was identified using a Vitek™ 2 automated instrument for infectious disease testing (bioMérieux SA, Marcy l’Etoile, France) for its resistance against imipenem and meropenem. Bacterial species were identified by the Vitek™ 2 automated instrument (bioMérieux); these species were subsequently confirmed using Matrix-Assisted Laser Desorption/Ionization Time of Flight Mass-Spectrometry (MALDI-TOF MS; Bruker Daltonics Inc., Bremen, Germany)^27^.

Additional analyses were performed to determine the clonal relationships between bacterial isolates identified in the first and second ESBL-E carriage testing. First, the Vitek™ 2 automated instrument was preliminary employed to test antimicrobial susceptibilities to 10 agents with potential activity against ESBL-E: tazobactam/piperacillin, cefmetazole, ceftazidime, cefepime, aztreonam, gentamicin, amikacin, ciprofloxacin, levofloxacin, and trimethoprim/sulfamethoxazole. When the minimal inhibitory concentrations against an antimicrobial agent differed more than fourfold among bacterial isolates, these strains were determined as having different susceptibilities. Bacterial isolates with three or more different antimicrobial susceptibilities were determined as different strains. In this manner, we identified strains with a similar pattern of antimicrobial susceptibility between the first and second ESBL-E carriage testing. Pulsed-field gel electrophoresis (PFGE) were then employed to characterize the clonal relationships among these strains^28^. Genomic DNA was digested with XbaI enzyme (Takara Bio, Otsu, Japan), and the resulting DNA fragments were separated by PFGE in a CHEF-DR III system (Bio-Rad, Hercules, CA, USA) with pulses ranging from 5.3 to 49.9 s for 19.7 h at 6 V/cm and 14˚C. The banding patterns were analyzed with UPGMA clustering method with CLIQS™ (Totallab Ltd, Newcastle, UK)^29^.

### Statistical analysis

All analyses were performed using IBM SPSS Statistics for Windows, Version 25 (IBM Corp., Armonk, NY, USA). Categorical variables were presented as number and percentage, while numerical variables were presented as median and range unless otherwise indicated. The chi-square test was used to compare ESBL-E carriage statuses between residents in Cohort 1 and Cohort 2 (Analysis Part 1) with a significance threshold of *p* < 0.05. All methods were performed in accordance with the Declaration of Helsinki. Jichi Medical University Bioethics Committee for Medical Research approved the study and waived the requirement for informed consent from individual participants (Approval ID: 20–058).

## Results

The total 260 residents comprised 29 (11.2%), 52 (20%), 89 (34.2%), and 90 (34.6%) residents from facilities A, B, C, and D, respectively.

### Analysis part 1

Cohort 1 and Cohort 2 contained 75 and 185 residents, respectively (Table 2). Among all 260 residents, 95 (36.5%) were positive for ESBL-E carriage in the first testing. Compared with the residents in Cohort 1, a significantly larger proportion of residents in Cohort 2 were positive for ESBL-E carriage (28.0% in Cohort 1 vs 40.0% in Cohort 2, *p* < 0.01). The ESBL-E strains identified in the first testing had considerable diversity (Table 3). Among 95 residents with ESBL-E carriage, a single strain of *Escherichia coli* was identified in 74 residents (77.9%). Only one strain met the definition of CRE; this strain produced ESBL plus AmpC β-lactamase.

**Table 2:** ESBL-E carriage statuses among residents in Cohort 1 and Cohort 2 (N = 260; Analysis Part 1).

	First ESBL-E test result^†^				Total	
	Positive		Negative			
	n	(%)	n	(%)	n	(%)
Cohort 1	21	(28.0)^‡^	54	(72.0)	75	(100)
Cohort 2	74	(40.0)^‡^	111	(60.0)	185	(100)
Total	95	(36.5)	165	(63.5)	260	(100)

ESBL-E, extended-spectrum β-lactamase-producing Enterobacterales.

^†^Performed < 1 month after study onset or initiation of residence.

^‡^Chi-square test: *p* < 0.01.

**Table 3:** Classification of ESBL-producing bacteria detected in fecal specimens from residents.

Bacteriological classification of ESBL-producing bacteria	N	(%)
**Carriage of a single strain**		
*Escherichia coli*	74	(77.9)
AmpC β-lactamase co-producing *Escherichia coli*	3	(3.2)
*Citrobacter freundii*	1	(1.1)
*Enterobacter cloacae*	1	(1.1)
*Proteus mirabilis*	1	(1.1)
**Carriage of a multiple strains**		
*Escherichia coli* + *Klebsiella pneumoniae*	7	(7.4)
*Escherichia coli* + *Klebsiella oxytoca*	2	(2.1)
Two strains of *Escherichia coli*	2	(2.1)
Three strains of *Escherichia coli*	1	(1.1)
*Escherichia coli* + AmpC β-lactamase co-producing *Escherichia coli*^†^	2	(1.1)
*Escherichia coli* + AmpC β-lactamase co-producing *Enterobacter cloacae*	1	(1.1)
Total	95	(100)

ESBL, extended-spectrum β-lactamase.

^†^One strain met the definition of carbapenem-resistant Enterobacteriaceae (CRE).

### Analysis part 2

**Figure 2:**
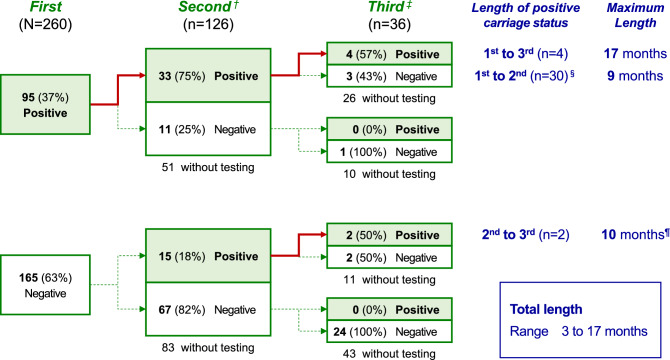
Lengths of positive ESBL-E carriage statuses among residents who underwent the second testing and third testing (n = 36; Analysis Part 3). ^†^Performed 3–12 months after the first testing. ^‡^Performed ≥ 12 months after the second testing. ^§^Excluding 4 residents with positive results in the third testing. ^¶^These two residents exceptionally underwent the third testing at 10 months after the second testing. ESBL-E, extended-spectrum β-lactamase-producing Enterobacterales.

Of the 126 residents who underwent both the first testing and second testing (Fig. 2; Table 4), 48 (38.1%) were positive for ESBL-E carriage in the second testing. Among the 44 residents who were positive for ESBL-E carriage in the first testing, 33 (75.0%) remained positive in the second testing. However, among the 82 residents negative for ESBL-E carriage in the first testing, 15 (18.3%) showed positive conversion in the second testing.

**Table 4:** Changes in ESBL-E carriage statuses from the first testing to second testing (n = 126; Analysis Part 2).

	Second ESBL-E test result^†^				Total (n = 126)	
	Positive (n = 48)		Negative (n = 78)			
	n	(%)	n	(%)	n	(%)
First ESBL-E test result						
Positive	33	(75.0)^‡^	11	(25.0)	44	(100)
Negative	15	(18.3)^§^	67	(81.7)	82	(100)

ESBL-E, extended-spectrum β-lactamase-producing Enterobacteriaceae.

^†^Performed 3–12 months after the first ESBL-E testing.

^‡^95% confidence interval: 59.4% to 86.3%.

^§^95% confidence interval: 10.9% to 28.7%.

The 2.5th and 97.5th percentiles were used to express 95% confidence intervals.

Of the 36 residents who underwent both the second testing and third testing (Fig. 2; Table 5), 6 (16.7%) were positive for ESBL-E carriage in the third testing. Among the 11 residents who were positive for ESBL-E carriage in the second testing, 6 (54.4%) remained positive in the third testing. Furthermore, among the 25 residents negative for ESBL-E carriage in the second testing, no residents showed positive conversion in the third testing.

**Table 5:** Changes in ESBL-E carriage statuses from the second testing to third testing (n = 36; Analysis Part 2).

	Third ESBL-E test result^a^				Total (n = 36)	
	Positive (n = 6)		Negative (n = 30)			
	n	(%)	n	(%)	n	(%)
Second ESBL-E test result						
Positive	6	(54.5)^b^	5	(45.5)	11	(100)
Negative	0	(0)	25	(100)	25	(100)

ESBL-E, extended-spectrum β-lactamase-producing Enterobacteriaceae.

^a^Performed ≥ 12 months after the second ESBL-E testing.

^b^95% confidence interval: 24.6% to 81.9%.

The 2.5th and 97.5th percentiles were used to express 95% confidence interval.

### Analysis part 3

The lengths of ESBL-E carriage in the residents who underwent the second testing and third testing are shown in Fig. 2. The lengths of carriage were measured for 4, 30, and 2 residents for the first testing to third testing, first testing to second testing, and second testing to third testing, respectively. The maximum length of ESBL-E carriage was 17 months.

**Figure 3:**
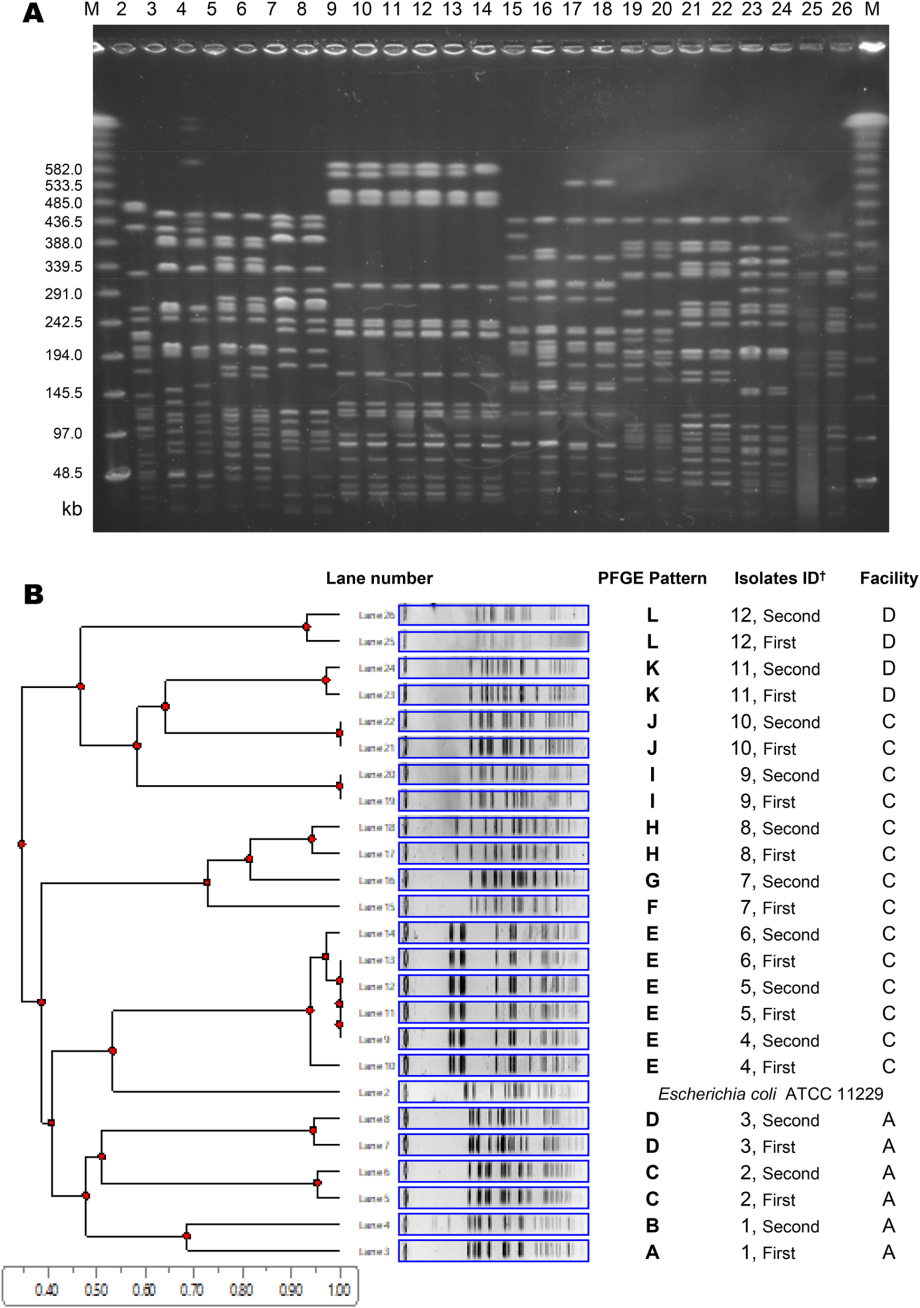
Pulsed-field gel electrophoresis typing. (**A**) Original pulsed-field gel electrophoresis (PFGE) profiles of *E. coli* strains isolated from 12 residents. Lanes 1 and 27, molecular weight marker; lane 2, *E. coli* ATCC 11,229; and lanes 3–26, *E. coli* strains isolated from 12 residents. (**B**) The dendrogram was created by UPGMA clustering method using the software CLIQSTM (Totallab Ltd), and PEGE patterns were automatically rearranged in a figure. ^†^Resident identification number and ESBL-E carriage testing (the first or second) *Note*: The figure could not show the results of all the 44 strains (22 pairs) altogether because of a limit of the analytic device (thus, shows selected 24 strains only).

Among 33 residents who were positive for ESBL-E carriage in both first and second testing, 22 (66.7%) were carriers with specific bacterial strains whose antimicrobial susceptibilities were similar between the first and second testing: 44 strains in 22 pairs (a pair comprised strains isolated in the first and second testing). All strains were *E. coli*. PFGE was subsequently employed to compare the clonality of these strains, resulting in the detection of 15 pairs with each similar PFGE pattern. Specific *E. coli* strains with a similar specific PEGE pattern were found in 3 different residents in Facility C (Fig. 3).

## Discussion

The present study had three key findings. First, the prevalence of ESBL-E carriage was significantly higher in Cohort 2 than in Cohort 1 (40% vs 28%), which indicates that ESBL-E was most likely to be carried into LTCFs through admission of new residents, who provided a large contribution to the increased total prevalence of ESBL-E carriage among LTCF residents. Second, 18% (15/82) of residents showed positive conversion to ESBL-E carriage in the second testing, which indicates that some residents may have acquired ESBL-E through potential cross-transmission inside the LTCFs after short-term residence (3–12 months after admission). Additional PFGE analysis indicated that 3 different residents in the same LTCF were ESBL-E carriers of a specific strain whose clonality was similar. Third, some residents maintained ESBL-E carriage for > 1 year (maximum, 17 months), which indicates that these residents may be high-risk triggers for outbreaks inside LTCFs through resident-to-resident transmission as a potential ESBL-E reservoir. However, we subsequently found that no residents exhibited positive conversion to ESBL-E carriage after long-term residence (> 12 months after admission). This finding indicated that, from the long-term perspective, residents with ESBL-E carriage were less likely to accumulate inside LTCFs. Thus, the present findings highlight the importance for appropriate implementation of practical ESBL-E infection control and prevention measures by care providers in geriatric LTCFs, with the expectation of disappearing ESBL-E from the facilities.

Using the exact same LTCF settings employed in the present study, we recently reported that the prevalence of methicillin-resistant *Staphylococcus aureus* (MRSA) in the nasal cavity of LTCF residents was approximately 10%^23^. However, the prevalence of ESBL-E carriage among residents was much higher in the present study (36.5%; 95/260 residents). We further reported that MRSA may be imported into LTCFs via transfer of residents rather than spread by potential cross-transmission inside LTCFs^23^. Similar to MRSA, the present study indicated that ESBL-E were most likely to be carried into LTCFs through admission of new residents. However, major differences between MRSA and ESBL-E may be the frequency of potential cross-transmission and the length of carriage. Compared with MRSA^23^, ESBL-E was more likely to become widespread inside geriatric LTCFs through potential cross-transmission among residents and less likely to disappear spontaneously after short-term residence. Our additional PFGE analysis results support potential cross-transmission among residents. These differences may contribute to the higher prevalence of ESBL-E compared with MRSA. Given that ESBL-E can be transmitted during the excrement disposal process or fecal contamination, care providers in geriatric LTCFs should pay thorough attention to adherence for infection prevention, especially for residents requiring diaper disposal.

The present results indicated that residents with ESBL-E carriage may diminish during long-term residence. This finding was supported by a previous study. Overdevest et al.^11^ conducted a surveillance study and suggested that ESBL-E could be predicted to disappear from LTCFs over time. They also reported that the lengths of ESBL-E carriage differed in accordance with the strain types, with ESBL-*Escherichia coli* of sequence type O25:ST131 having the longest carriage period before its disappearance from LTCFs^11^. In the present study, ESBL-*Escherichia coli* was only identified in residents with prolonged ESBL-E carriage for > 12 months (data not shown), although we did not identify sequence types. We further found that 17% of residents had acquired ESBL-E through potential cross-transmission inside the LTCFs within short-term residence after admission. Although there are no previous studies to support this finding, some possible hypotheses can be proposed. First, new residents admitted to LTCFs may have had multiple risk factors for ESBL-E acquisition, such as episodes of recent antibiotic use and/or previous hospitalization^12–15,18,19,30,31^. Our selected LTCFs had their own linkages with specific back-up hospitals. Second, ESBL-E may have been most infectious immediately after being carried into the facilities, and then gradually become less infectious. This may be associated with the duration from previous antibiotics use. Importantly, our findings highlight that the risk of ESBL-E acquisition inside geriatric LTCFs may further increase with admission of large numbers of new residents.

Care providers in geriatric LTCFs should consider that residents are most likely to have ESBL-E in their feces on their initial admission to the facilities, especially those admitted from hospital settings. Furthermore, residents with short-term residence should be considered as high-risk residents for ESBL-E acquisition. A patient traceability with alarm system for ESBL-E carriage between specific back-up hospitals and their receiving LTCFs may be effective in preventing ESBL-E transmission inside LTCFs. However, the standard precautions, such as thorough hand hygiene and appropriate use of gloves, should be of the greatest importance for all care providers in geriatric LTCFs. A previous study showed that enhanced infection control measures mainly based on thorough adherence to standard precautions led to subsidence of an outbreak of ESBL-producing bacteria inside an LTCF^32^. Several intervention programs may improve adherence to standard precautions among care providers in geriatric LTCFs^2,3,33^. In particular, care providers should be careful about daily care in the excrement disposal process and fecal contamination for residents requiring diapers. Such residents comprised the majority in our study settings. To our knowledge, no previous studies have focused on older adults with diapers and infections. This situation may warrant further assessment of infection control and prevention strategies against ESBL-E.

The primary limitation of the present study was incomplete information on the background characteristics of the residents, such as sex, age, general condition, medical history. Among these factors, general condition (activities of daily living and nutritional status) and medical history (comorbidities, use of antimicrobials, and duration of prior hospitalization) may affect the prevalence of ESBL-E carriage^3,6,13,30^, which would result in potential confounders of our findings. Unfortunately, the institutional ethics review board did not grant approval to collect this information without obtaining written informed consent from each resident even though the information was anonymized. To confirm the present findings, further studies should be conducted with complete information for residents, including potential factors associated with ESBL-E carriage status. Second, a large number of residents did not undergo the second testing and third testing. These residents could not undergo the second testing and third testing because they had been already discharged from the LTCFs at the time of the testing. Third, we performed ESBL-E testing only three times with different intervals. Multiple tests with regular intervals and longer follow-up may be required to accurately assess the status of ESBL-E carriage. Fourth, we were unable to obtain information on the prevalence of ESBL-E carriage in region-specific general populations surrounding each LTCF and among patients hospitalized in each specific back-up hospital, which may have affected our results. Fifth, the study participants comprised a small number of residents from a limited number of LTCFs. Studies with larger numbers of residents from a greater number of LTCFs located in various regions (both rural and urban areas) are warranted to further confirm our findings. Sixth, we could not identify sequence types of specific bacterial strains. Finally, a standard infection prevention and control protocol was not formally established in all four LTCFs during the study period, which may have affected the prevalence of ESBL-E carriage.

## Conclusions

ESBL-E was most likely to be introduced into LTCFs through admission of new residents. Furthermore, some residents acquired ESBL-E through potential cross-transmission inside the LTCFs within a short period (3–12 months) after admission. Although ESBL-E carriage could be maintained for > 1 year, no residents exhibited positive conversion to ESBL-E carriage upon long-term residence of > 12 months after admission, which indicates that residents with ESBL-E carriage may not accumulate inside LTCFs. Practical infection control and prevention by care providers could decrease the prevalence of ESBL-E in geriatric LTCFs.
